# Ultimately short ballistic vertical graphene Josephson junctions

**DOI:** 10.1038/ncomms7181

**Published:** 2015-01-30

**Authors:** Gil-Ho Lee, Sol Kim, Seung-Hoon Jhi, Hu-Jong Lee

**Affiliations:** 1 Department of Physics, Pohang University of Science and Technology, Pohang 790-784, Republic of Korea; 2 Present address: Department of Physics, Harvard University, Cambridge, Massachusetts 02138, USA

## Abstract

Much efforts have been made for the realization of hybrid Josephson junctions incorporating various materials for the fundamental studies of exotic physical phenomena as well as the applications to superconducting quantum devices. Nonetheless, the efforts have been hindered by the diffusive nature of the conducting channels and interfaces. To overcome the obstacles, we vertically sandwiched a cleaved graphene monoatomic layer as the normal-conducting spacer between superconducting electrodes. The atomically thin single-crystalline graphene layer serves as an ultimately short conducting channel, with highly transparent interfaces with superconductors. In particular, we show the strong Josephson coupling reaching the theoretical limit, the convex-shaped temperature dependence of the Josephson critical current and the exceptionally skewed phase dependence of the Josephson current; all demonstrate the *bona fide* short and ballistic Josephson nature. This vertical stacking scheme for extremely thin transparent spacers would open a new pathway for exploring the exotic coherence phenomena occurring on an atomic scale.

## Abstract (web summary)

The functionality of Josephson junctions—a thin insulating layer between two superconducting regions—is greatly enhanced by using hybrid material systems. Here, the authors incorporate graphene into a Josephson junction and demonstrate ballistic transport through the atom-thick layer.

## Graphical abstract

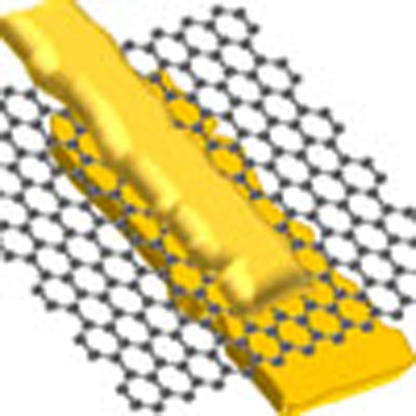

Diverse nanometre-scale hybrid superconducting devices^1^,^2^, that is, two superconductors (S) coupled by a normal-conducting (N) insert, have been utilized for field-effect supercurrent transistors^3^,^4^,^5^, quantum electron pumps^6^, gate-tuneable quantum systems^7^ and, more recently, hybrid topological-insulator systems in search for the Majorana fermionic excitation states^8^,^9^. However, these efforts have often been hindered by non-ideal S–N contact characteristics and weak superconducting coherence, attributed to the short electronic mean free path (*l*) and/or a short superconducting coherence length (*ξ*), compared with the channel length (*L*) between the two superconducting electrodes.

A promising scheme for realizing short (*L*<*ξ*) and ballistic (*L*<*l*) Josephson junctions (JJs) is combining superconductors with the two-dimensional electron gas (2DEG) in semiconductor heterostructures with remarkably long *l* (=10–100 μm). However, detrimental etching processes, required to access the 2DEG layer imbedded deep inside the heterostructure or to remove the native surface oxides, seriously reduces *l* of the contact^10^ down to ~10 nm. This results in the diffusive behaviour of the JJs, although the junction channel retains the ballistic character^11^. Recently, graphene has been adopted for JJ fabrication^5^,^7^,^12^, where charge carriers behave as massless chiral relativistic particles with Cooper pair formation between time-reversal symmetric carriers in opposite valleys. Even for graphene, with high carrier mobility and good superconducting contacts, however, a graphene-based JJ remains diffusive in nature. The major obstacle is that the junction channel length (*L*=30–300 nm), in conventional planar geometry, cannot be arbitrarily shortened but is limited by the resolution of the electron (e)-beam nanofabrication method^3^,^4^,^5^,^7^,^13^. In atomic break junctions^14^,^15^, *L* can be reduced to the atomic scale. But the limited number of conducting channels in the break junction leads to substantially weakened Josephson coupling, making the junction prone to enhanced fluctuations^14^, both quantum and classical. In addition, independent control over multiple atomic constrictions on a single substrate would be unviable, making it difficult to scale up the devices for practical applications.

Here we demonstrate the fabrication and transport characterization of highly transparent vertical graphene-based JJs with atomic-scale channel length and the scalability along lateral directions. Our devices show the short ballistic Josephson nature with strong Josephson coupling reaching the theoretical limit, the temperature dependence of the Josephson critical current and the phase dependence of the Josephson current that are consistent with the theoretical prediction.

## Results

### Vertical graphene Josephson junctions

**Figure 1:**
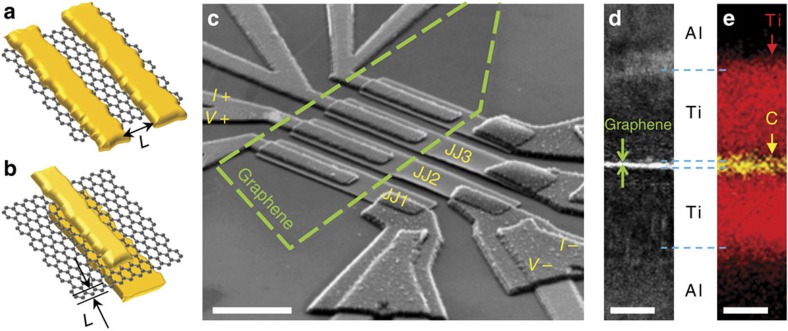
Vertical graphene Josephson junction. (**a**) In a planar-junction geometry, the reduction of the junction length, *L*, is limited by the roughness of the electrode edges. (**b**) In a vertical-junction geometry, *L* is replaced by the thickness of a single graphene layer, even with rough electrode edges. (**c**) Scanning electron microscopy image of four nominally identical vertical graphene Josephson junctions (vGJJs). The behaviour of the junction JJ2 was described in detail in the text; MAR was measured in the other junctions (JJ1 and JJ3) to estimate superconducting gap. Monolayer graphene, whose boundary is denoted by a green-dashed line, is sandwiched between the top and bottom Ti/Al/Au superconducting electrodes. In a four-probe measurement setup, the current was biased between *I*+ and *I*−, along with simultaneous measurements of the voltage drop between *V*+ and *V*−. Scale bar, 5 μm. (**d**) High-resolution bright-field spherical-aberration-corrected scanning transmission electron microscopy (STEM) image of the cross-section of a vGJJ. The monolayer graphene sheet was atomically in contact with the titanium layer. The highest-intensity peak at the interface corresponded to a width of ~0.44±0.01 nm, which was identical to the thickness of monolayer graphene. Scale bar, 5 nm. (**e**) Electron energy loss spectroscopy image of the same area as the STEM image in **d**. Red (yellow) colour denotes the titanium (carbon) element. The monolayer graphene consisting of carbon atoms was sandwiched by two 8-nm-thick titanium adhesion layers. Scale bar, 5 nm.

In this study, we used defect-free and single-atom-thick exfoliated graphene as a normal-metallic insert for a graphene JJ (Fig. 1c) in a vertical geometry (Fig. 1b) to overcome both the lithographical limitations in planar geometry (Fig. 1a) and poor scalability of the atomic break junctions (see Methods for fabrication details). Scanning transmission electron microscopy (Fig. 1d) and electron energy loss spectroscopy (Fig. 1e) images of the same junction cross-section confirmed that the monolayer graphene formed an atomically sharp interface with the Ti adhesion layers. Indeed, vertical graphene JJs (vGJJs) have an atomically short channel length of *L*=0.4 nm, corresponding to the thickness of a single graphene layer, with scalable lateral dimensions (Supplementary Fig. 1). In addition, the excellent chemical inertness of graphene and e-beam nanofabrication for direct deposition of metallic electrodes on both sides of the graphene, combined with the ‘flip-transfer’ technique^16^, allowed achieving almost perfect transparency of the S–N contacts.

### Strong Josephson coupling in vertical graphene Josephson junctions

**Figure 2:**
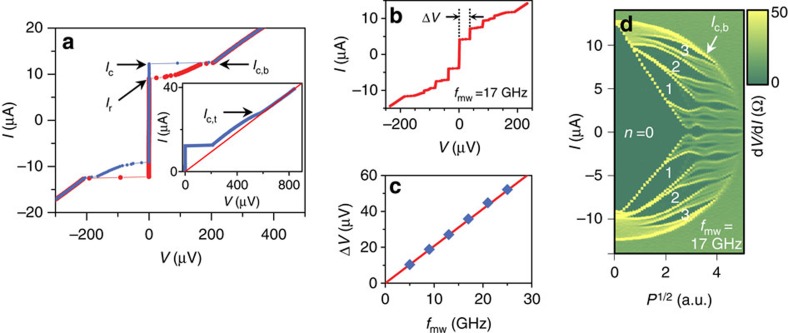
Josephson coupling through graphene. (**a**) Current–voltage (*I–V*) characteristics of the junction JJ2, measured at the base temperature of 50 mK with a current sweep from negative to positive (blue dots) and vice versa (red dots), exhibited hysteretic behaviour, which may have been of thermal origin^38^. Critical currents of the JJ (*I*_c_), the bottom electrode (*I*_c,b_), the top electrode (*I*_c,t_) and the retrapping current (*I*_r_) are denoted by arrows. *I*_c_ and *I*_c,b_ coincide with each other at the base temperature. (Inset) *I–V* characteristics using an expanded scale show the critical current of the top electrode (*I*_c,t_), above which the *I–V* characteristics exhibited linear behaviour, represented by the red line passing through the origin with normal-state resistance, *R*_N_. *I*_c,b_ and *I*_c,t_ may have been decreased by self-heating in the junction, because they appeared after the JJ switched to the resistive state, whereas *I*_c_ itself was free from self-heating. (**b**) Shapiro steps under microwave exposure of frequency *f*_mw_=17 GHz and amplitude *P*^1/2^=1.8 (a.u.), occurring in steps of 35.8 μV. (**c**) Measured Δ*V* under microwaves of various *f*_mw_ (symbols) showed good agreement with the ac Josephson relation of Δ*V*=(*h*/2*e*)*f*_mw_ (line). (**d**) Colour-coded plot of d*V*/d*I* as a function of the bias current and *P*^1/2^ at a fixed frequency of *f*_mw_=17 GHz; higher-order Shapiro steps were observed.

Below the superconducting critical temperature of the electrodes (*T*_c,b_=0.75 K and *T*_c,t_=1.00 K for the bottom and top electrodes, respectively), the proximity effect^1^,^2^ induces superconductivity in the graphene layer along with Josephson coupling, represented by the current–voltage (*I*–*V*) characteristics of vGJJ (JJ2) in Fig. 2a. As the bias current increased, the zero-resistance supercurrent state abruptly jumped to the resistive state at the junction critical current of *I*_c_=13.3 μA. Above the critical currents of both electrodes (*I*_c,b_ and *I*_c,t_ for the bottom and top electrodes, respectively), the *I*–*V* curves became linear crossing the origin with the normal-state resistance of *R*_N_=21.4 Ω (inset of Fig. 2a). We will see in Fig. 5 that, according to the first-principle calculation for the atomic structure of vGJJ, a potential barrier emerges at a graphene/Ti interface, which makes a main contribution to *R*_N_. Whereas, Ti/Al metal-to-metal interfaces are highly transparent and lead to a very low *R*_N_ (~0.1 Ω) in an Al/Ti/Al junction of a control experiment (see Methods).

The *I*_c_*R*_N_ product is an important junction parameter that represents the characteristic quality of a JJ, without the sample-specific geometrical factors^2^. The *I*_c_*R*_N_ product reaches 285 μV, corresponding to 2.59*Δ*_0_/*e*, with superconducting gap *Δ*_0_=110 μeV determined by measurements of the multiple Andreev reflection (MAR; see Methods). Here, *Δ*_0_ (=2*Δ*_t_*Δ*_b_/(*Δ*_t_+*Δ*_b_)) is the combined superconducting gap energy^17^ for the unidentical gap energy of top (*Δ*_t_) and bottom (*Δ*_b_) aluminium electrodes. The normalized value of *eI*_c_*R*_N_/*Δ*_0_ (=2.59) exceeds the theoretical limit [(*eI*_c_*R*_N_/*Δ*_0_)_max_=2.07] for a JJ in the short (*L*<*ξ*) and diffusive (*L*>*l*) regime^1^,^18^, despite the fact that the observed *I*_c_ may have been underestimated owing to the quantum and thermal fluctuations^2^, and the instrumental noise. The normalized *I*_c_*R*_N_ product is also in sharp contrast to the maximal values reported for superconductor–normal conductor–superconductor (S–N–S) proximity JJs incorporating 2DEG (ref. 19) (~0.9), single-crystalline nanowires^20^ (~1.5), graphene^5^ (~1.0), suspended graphene^12^ (~0.3) and graphene-based superconducting quantum interference device (SQUID)^21^ (~1.0), all of which are substantially below the upper limit (2.07) for a short diffusive JJ. The large *I*_c_*R*_N_ product of our vGJJ, in sharp discord with the short diffusive character, is explicable by the short ballistic character^1^,^22^ (*L*<*ξ*, *l*) of the junction, where the value of *eI*_c_*R*_N_/*Δ*_0_ can reach *π* maximally but is reduced depending on the *I*_c_-reduction factor *α* introduced below. It would be a subtle issue to consider the atomic vertical transport of vGJJ in terms of the mesoscopic parameters such as *l* and *ξ*. vGJJ turns out to be a unique system in which its vertical transport is microscopic in nature while the in-plane dimensions are in mesoscopic scales. Here, we extend the well-established mesoscopic theory of proximity JJs to describe vGJJ as an extreme case of atomically short *L*. As shown later, this approach successfully describes the observed features of vGJJs with short ballistic characters of JJs. We consider the Ti layers as parts of the superconducting electrodes with the proximity-induced gap that is the same as that of adjacent Al layers, since Ti layers are much thinner than *ξ* in Ti, ~140 nm (see also the solutions for the Usadel equation in Discussion). The dependence of *I*_c_*R*_N_ product on sample-specific contact characteristics is also discussed in Supplementary Fig. 2.

Applying microwave irradiation of frequency *f*_mw_ results in a series of quantized voltage plateaus (known as Shapiro steps^2^) at *V*_*n*_=*nhf*_mw_/2*e* in the *I*–*V* curves, as shown in Fig. 2b (*n* is an integer and *h* is Plank’s constant). The appearance of Shapiro steps by the ac Josephson effect rigorously confirms that the supercurrent originated from Josephson coupling, rather than artefacts such as electrical shorting across the junction. In addition, the in-plane magnetic field dependence of *I*_c_, known as the Fraunhofer pattern^2^, further supports the establishment of genuine Josephson coupling (Supplementary Fig. 3). In Fig. 2c, the voltage step height, Δ*V*, and *f*_mw_ in the range of 5–25 GHz showed good agreement with the expected linear relationship, Δ*V*=(*h*/2*e*) *f*_mw_. Shapiro steps, represented as zero differential resistance (d*V*/d*I*=0) in Fig. 2d, exhibited quasi-periodic oscillations with increasing microwave amplitude *P*^1/2^ in the current range bounded by *I*_c,b_ (<*I*_c,t_), above which the bottom electrode becomes normal, losing Josephson coupling along with disappearance of the Shapiro steps. Figure 2c also reveals fractional Shapiro steps for n*=*n*/2 and *n*/3, which implies a nonsinusoidal current–phase relation (CPR) for the junction, which will be further discussed later (see also Supplementary Fig. 4).

### Temperature dependence of the critical current

**Figure 3:**
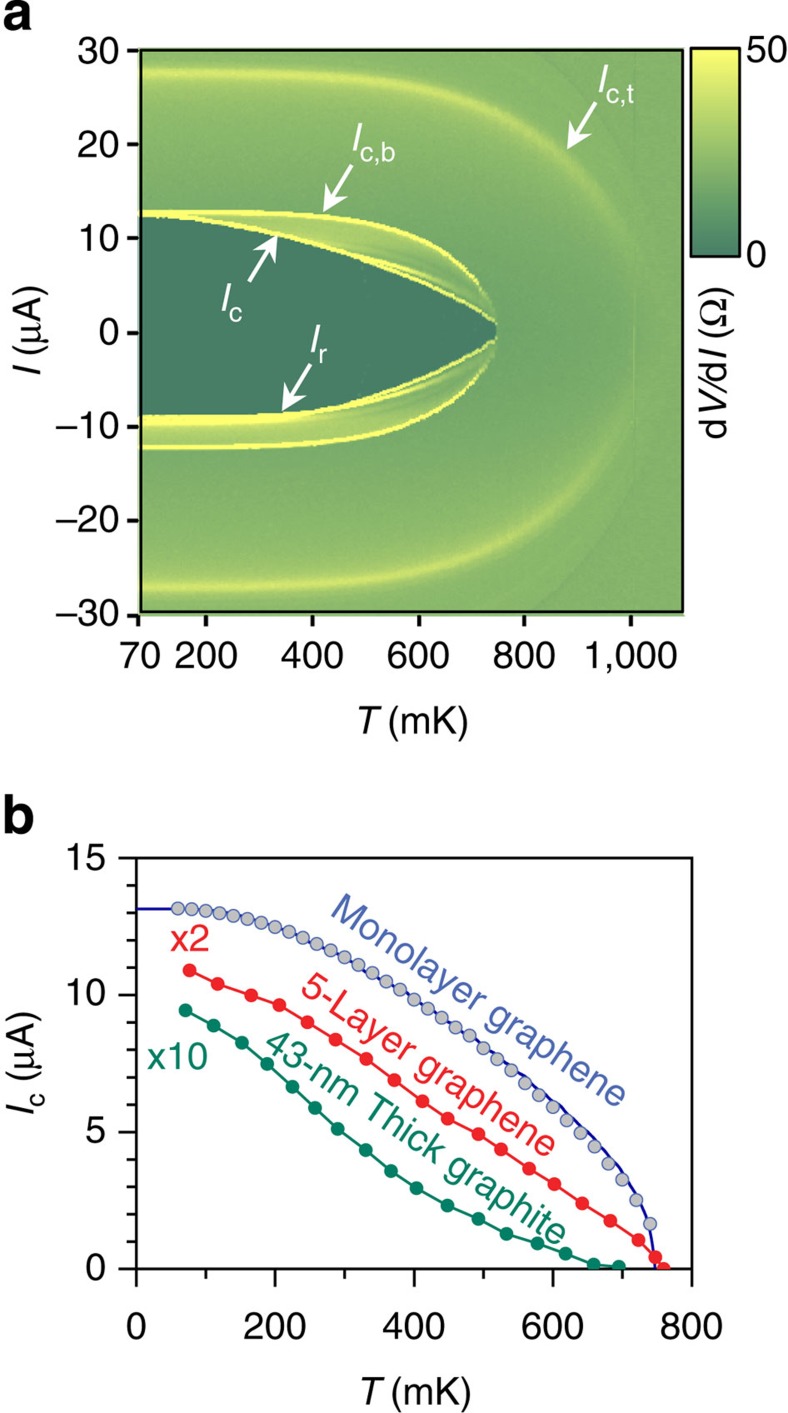
Temperature dependence of the junction critical current. (**a**) Colour-coded plot of d*V*/d*I*, measured with a current sweep from negative to positive as a function of temperature *T*. *T*_c,b(t)_ was determined as the temperature where the critical current *I*_c,b(t)_ vanished at the interface between the bottom (top) electrode and graphene. Above the critical temperatures of *T*_c,b_ and *T*_c,t_ at the bottom and top electrodes, respectively, d*V*/d*I* became equal to the normal-state resistance, *R*_N_. (**b**) Experimentally measured *I*_c_ (blue symbols) of monolayer graphene vGJJ (JJ2), along with the best-fit curve to the short ballistic junction characters (blue line). Temperature dependences of *I*_c_ for vGJJs made of five-layer graphene (red symbols) and 43-nm-thick graphite (green symbols). Lines are provided as guides.

Having established strong Josephson coupling in the vGJJ, we now discuss its temperature (*T*) dependence in detail. The *T* dependence of *I*_c_, identified as the bright local maximum curves of d*V*/d*I* in Fig. 3a, showed two uncommon features. One is that *I*_c_ appeared immediately below the electrode critical temperatures (in this case, *T*_c,b_), that is, the junction critical temperature (*T*_c,JJ_) is identical to *T*_c,b_, indicating the establishment of strong Josephson coupling in the vGJJ. In ordinary proximity JJs, *T*_c,JJ_ is noticeably below the critical temperature of the electrodes (Δ*T*~0.3 K, ~0.1 K and ~1.6 K for Nb-2DEG-Nb (ref. 19), Al-nanowire-Al^20^ and Pb-graphene-Pb JJs^23^, respectively) because sufficiently strong superconductivity of the electrodes is required for discernible Josephson coupling to be established. Another uncommon feature is that *I*_c_ decreases with a convex-shaped *T* dependence, that is, d^2^*I*_c_(*T*)/d*T*^2^<0, up to *T*_c,JJ_. Whereas, diverse proximity JJs studied to date have shown a concave-shaped or exponentially decaying *I*_c_(*T*) for *T* close to *T*_c,JJ_, which is a typical long-junction behaviour. As *L* is increased with thicker graphite flakes, the convex-shaped *I*_c_(*T*) gradually changed to a concave-shaped or exponential decay (Fig. 3b and Supplementary Fig. 5). The convex-shaped *I*_c_(*T*), especially close to *T*_c,JJ_, was predicted uniquely for JJs in the short-junction limit^22^ in the 1970s; however, its experimental observation has seldom been reported except for a few recent reports in high-*T*_c_ edge JJs, although values of the *I*_c_*R*_N_ product of the junctions fall far below the gap value.

For more compelling evidence for the short ballistic character of vGJJ, we performed a quantitative analysis on *I*_c_(*T*). From a microscopic viewpoint, the Josephson current in the ballistic regime is carried by discrete energy states of Andreev-reflected coherent electron–hole pairs, referred to as an Andreev-bound state (ABS)^24^. This ABS has recently been experimentally demonstrated as a new test bed for quantum information devices, that is, the Andreev-level qubit^25^. For *L*<*ξ*, Josephson coupling is established by a single pair of ABS per conducting channel in the graphene layer, with an energy of 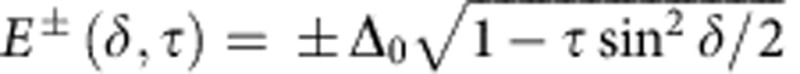, where *τ* is the junction transparency, and *δ* is the macroscopic quantum phase difference between the two superconducting electrodes. Here, *τ* is the ensemble-averaged transparency out of ~*R*_Q_/*R*_N_~10^3^ conducting channels, representing the overall behaviour of the junction (*R*_Q_=*h*/*e*^2^ is the quantum resistance). In thermal equilibrium at *T*, the Josephson current is given by 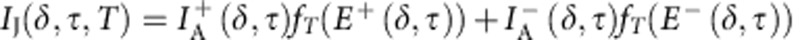, where 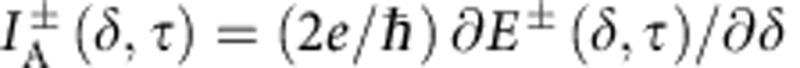 is the Josephson current carried by an ABS pair and 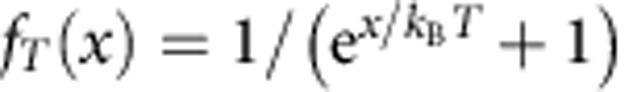 is the Fermi–Dirac distribution function (*ħ*=*h*/2*π* and *k*_B_ is Boltzmann constant). Consequently, the CPR of a short ballistic JJ is given by:


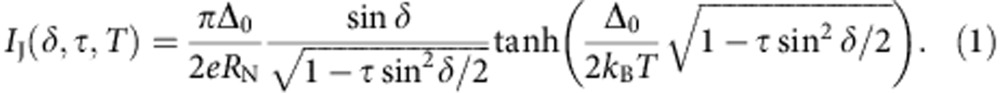


In a current-biased configuration, *δ* can have an arbitrary value, where *I*_c_ corresponds to the maximum value of *I*_J_ with respect to *δ*, that is, 
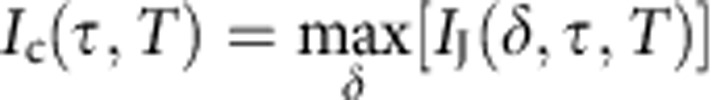
. As shown in Fig. 3b, the short ballistic character (black solid curve), obtained from equation (1), is in excellent agreement with the experimental data (symbols), showing a convex-shaped *I*_c_(*T*) with two best-fit parameters: *τ* (=0.98) and *α* (=0.93). High transparency almost reaching the ideal value of *τ*=1 reflects the high quality S–N interfaces in our device. Here, *α* parameterizes the reduction of measured *I*_c_ compared with the theoretical limit. The 7% reduction can be accounted for by premature switching due to fluctuations^2^ and/or instrumental noise, the Fermi-velocity mismatch at the S–N interfaces or the inverse proximity effect on the electrodes by graphene^26^.

### Current-phase relation measurements

**Figure 4:**
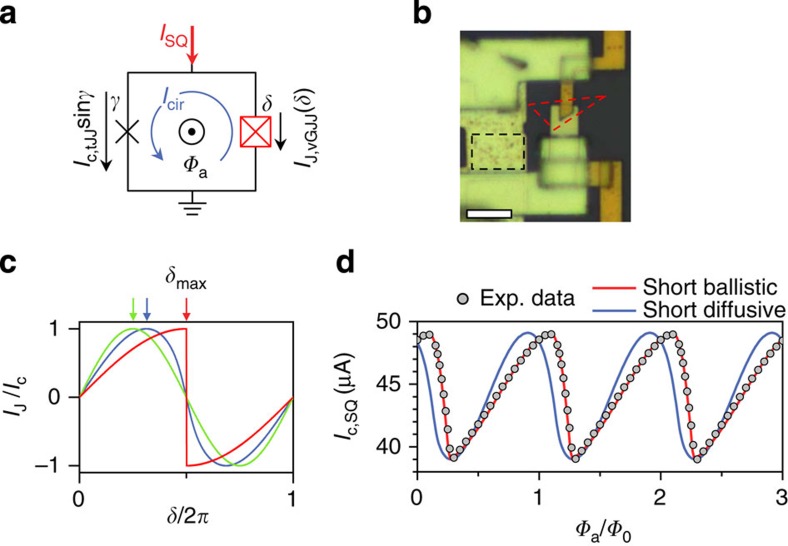
Measurements of the current-phase relation. (**a**) Schematic diagram of a dc-superconducting quantum interference device (SQUID) containing a tJJ of phase difference *γ* and a vGJJ of phase difference *δ* in a superconducting loop. (**b**) Optical micrograph from the SQUID interferometer used in this study. The tJJ and monolayer graphene are denoted by black- and red-dotted lines, respectively. Scale bar, 5 μm. (**c**) A highly skewed CPR for an ideal short ballistic JJ with *τ*=1 (*δ*_max_=*π*; red line), a skewed CPR for a short diffusive JJ (*δ*_max_=0.63*π*; blue line), and a sinusoidal CPR for tJJs with *τ*=0 (*δ*_max_=*π*/2; green line). *δ*_max_, denoted by an arrow for each case, represents the phase difference at which *I*_J_ is maximized. (**d**) Experimentally measured magnetic field dependence of the critical current of the SQUID at the base temperature, *I*_c,SQ_ (symbols), was in very good agreement with the expected variation calculated by the short ballistic theory of a JJ (red line) with fitting parameter *τ*=0.99. The theory for a short diffusive JJ (blue line) cannot account for the highly skewed experimental data.

For a rigorous confirmation of the short ballistic nature of the vGJJ and its potential application to Andreev-level qubits, it is essential to directly measure the CPR, *I*_J_(*δ*), which is closely related to the energy spectrum of the ABS. According to equation (1), a short ballistic JJ with *τ*=1 has a highly skewed nonsinusoidal CPR (red curve in Fig. 4c), distinctively different from the CPR of a short diffusive JJ (blue curve; calculated from the Usadel equations given in ref. 18) and the sinusoidal CPR of a conventional tunnelling JJ (tJJ) in the limit of *τ*=0 (green curve). Since the current-bias measurement is not sufficient for the CPR measurements, we performed phase-sensitive SQUID interferometry with varying *δ*, which captured the essence of the microscopic processes related to Josephson coupling^15^. The CPR measurement setup was based on an asymmetric dc-SQUID (Fig. 4a), which consisted of an Al/AlO_*x*_/Al tJJ of phase difference *γ* as a reference junction and a vGJJ as the subject junction under investigation of CPR [*I*_J,vGJJ_(*δ*)], imbedded in a superconducting loop. The phase difference in the vGJJ, imposed by the relationship *δ*=*γ*+2*πΦ*_a_/*Φ*_0_, was controllable by an external magnetic flux, *Φ*_a_, threading the SQUID loop (see Methods for the screening effect correction). Here, *Φ*_0_=*h*/(2*e*) is the magnetic flux quantum. In the limit of a much larger critical current for the tJJ (*I*_c,tJJ_), compared with that of the vGJJ (
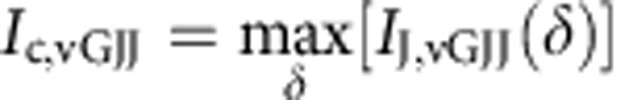
), the critical current of the SQUID, *I*_c,SQ_=max[*I*_c,tJJ_sin*γ*+*I*_J,vGJJ_(*δ*)], is dominated by the tJJ for *γ*~*π*/2, such that *I*_c,SQ_(*Φ*_a_)~*I*_c,tJJ_+*I*_J,vGJJ_(2*πΦ*_a_/*Φ*_0_+*π*/2). Thus, in principle, the magnetic flux dependence of *I*_c,SQ_ directly represents the CPR of the vGJJ.

In the SQUID interferometer shown in Fig. 4b, the tJJ was designed to have a much larger *I*_c,tJJ_ (=44.1 μA) than *I*_c,vGJJ_ (=5.1 μA). *I*_c,SQ_ as a function of *Φ*_a_ (symbols in Fig. 4d) exhibited a highly asymmetric, skewed CPR of the vGJJ, which was well described by the best-fit *I*_c,SQ_(*Φ*_a_) for a short ballistic JJ calculated using equation (1) (red curve), with the fitting parameter *τ*=0.99. However, the short diffusive character (blue curve) could not account for the high skewedness of the observed *I*_c,SQ_(*Φ*_a_). Thus, our CPR measurements provided irrefutable evidence for the short ballistic nature of vGJJs and the spectrum of the ABS. Qualitatively, the same behaviour was consistently obtained from other vGJJs prepared in a similar fashion (Supplementary Fig. 6).

### Calculation for the potential barriers at the interfaces

**Figure 5:**
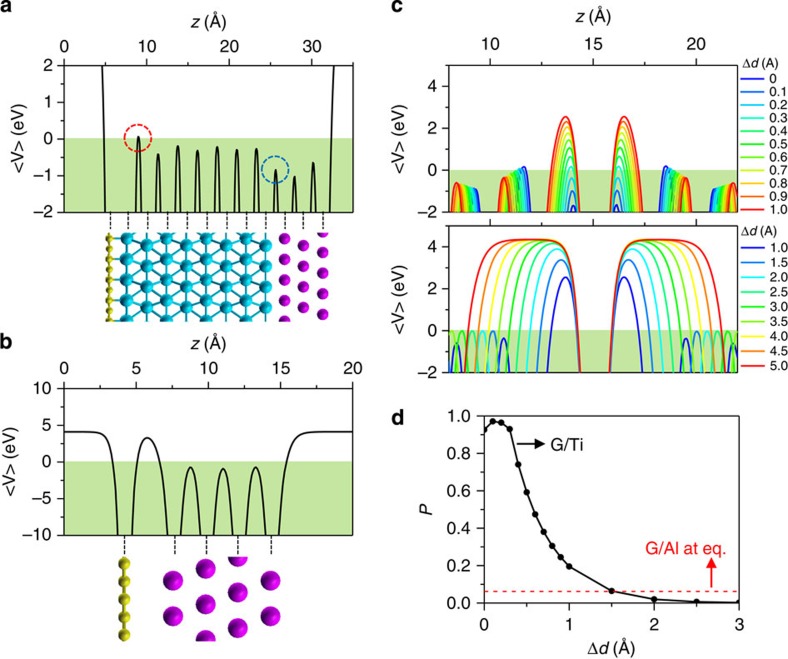
Calculation of interfacial potential barriers. (Upper panels) Electrostatic potential ‹*V*›(*z*) averaged over the *xy* plane (**a**) for graphene (G)/titanium (Ti)/aluminium(Al), and (**b**) for G/Al. The Fermi level is adjusted to zero. (Lower panels) Atomic structure of G (yellow)/Ti (blue)/Al (purple). (**c**) ‹*V*›(*z*) at Ti/G/Ti structure with varying distance between G and Ti layers (upper panel) for Δ*d*=0–1.0 Å in steps of 0.1 Å and (lower panel) for Δ*d*=1.0~5.0 Å in steps of 0.5 Å. (**d**) Numerical calculations of quantum tunnelling probability *P* through the potential barrier at G/Ti interface as a function of Δ*d*. Red dashed line represents *P* through the potential barrier of direct interface between G and Al layer.

To examine the characteristics of the interfaces, we performed the first-principles calculations using Vienna *Ab Initio* Simulation package^27^. We adopted the generalized gradient approximation^28^ augmented with the Tkatchenko–Scheffler van der Waals correction^29^ for the exchange-correlation of electrons. The cutoff energy for the plane wave-basis expansion was set to be 400 eV. The atomic relaxation was continued until the Helmann–Feynman forces acting on the atoms were <0.01 eV Å^−1^ (ref. 30). Here, to investigate potential barriers, if any, that emerge at graphene (G)/Ti and Ti/Al interfaces, we modelled atomic structure of vGJJ that consisted of 2 × 2 unit cell of graphene/eight-atom-thick Ti(0001)/three-atom-thick Al(111) slabs stacked along the *z* axis (visualized in the lower panel of Fig. 5a), and the vacuum region between the slabs was set to 15 Å. The grid for Brillouin zone sampling was set to 15 × 15 × 1 for relaxation and to 31 × 31 × 1 for charge density calculation. Calculated electrostatic potential ‹*V*›(*z*) averaged over the *xy* plane is shown in the upper panel of Fig. 5a with the same length scale as in the lower panel. There appears a narrow potential barrier (red dotted circle) above the Fermi level with height (*U*) of ~170 meV and width (*s*) of 0.22 Å. The barrier is located between the first and the second Ti layers. This is mainly owing to the expansion of Ti atomic distance as a result of the charge transfer from Ti to G. On the other hand, at the Ti/Al interface (blue dotted circle), there is no potential barrier, which implies an ideal contact between two metallic materials. In the case of G/Al interface in Fig. 5b, however, there appears a much larger and wider potential barrier than that in G/Ti. This justifies that Ti constitutes an adhesion layer between graphene and aluminium electrodes. For a more detailed analysis of the potential barrier at G/Ti interface, we investigated dependence of ‹*V*›(*z*) on the distance (*d*) between G and Ti slabs in terms of Δ*d*≡*d*–*d*_eq_ with *d*_eq_ (=2.24 Å), the equilibrium distance (Fig. 5c). As Δ*d* increases, the potential barrier between the first and the second Ti layers gradually decreases and vanishes above Δ*d*~0.3 Å, but a new barrier appears at the G/Ti interface with *U* saturating to the workfunction of Ti (~4.3 eV). At sufficiently low temperature (*k*_B_*T*~4 μeV≪*U*) and low bias voltage (*V*_b_~10 μeV≪*U*), electronic transport though the potential barrier solely arises from the quantum tunnelling. With slowly varying potential *U*(*z*), tunnelling probability *P* can be numerically calculated using Wentzel–Kramers–Brillouin (WKB) approximation as:^31^


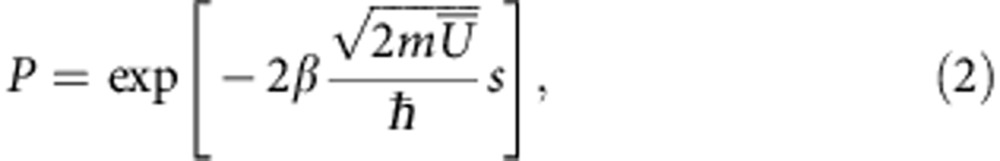


where the average potential is:


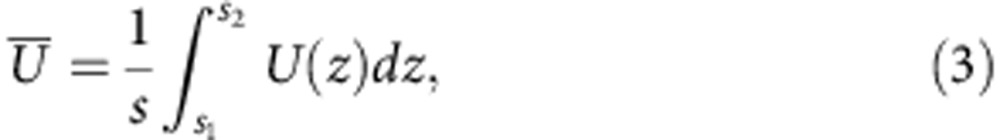


the dimensionless correction factor is:


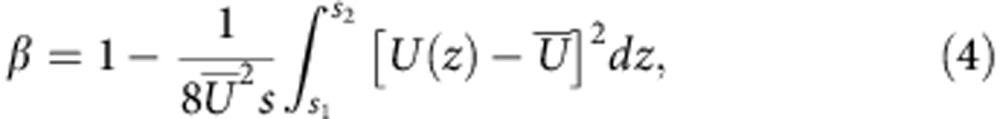


and *m* is the electron mass. *s*_1_ And *s*_2_ represent the start and the end point of the potential, respectively. In Fig. 5d, calculated *P* exhibits an exponential decay (black symbols) with Δ*d*. However, it is close to unity near the equilibrium point (Δ*d*<0.3 Å), which indicates the ohmic transport through the barrier with high transparency rather than tunnelling conduction. This supports that the vGJJ behaves as a S–N–S JJ, rather than a tJJ. Compared with transparent G/Ti interface, G/Al shows much suppressed *P*~0.1 (red dotted line in Fig. 5d).

## Discussion

**Figure 6:**
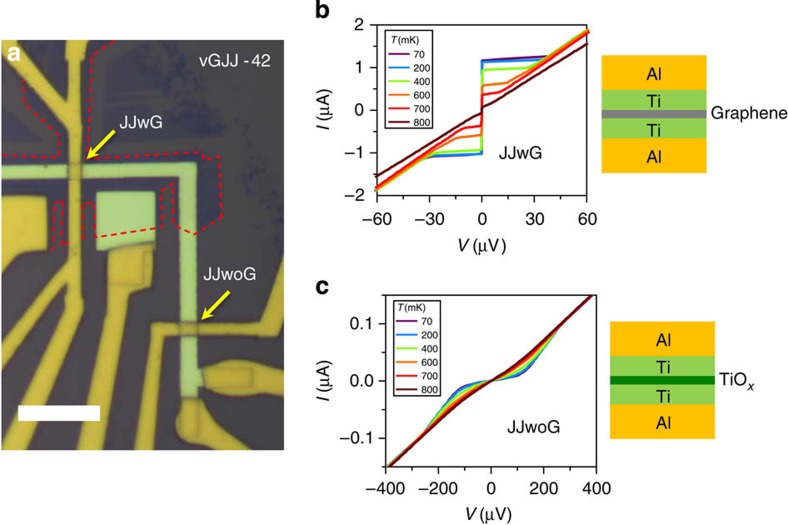
Vertical graphene Josephson junctions with and without graphene insertion. (**a**) Optical image of vGJJ devices with graphene (JJwG) and without graphene (JJwoG). Bottom electrode looks brighter than yellowish-coloured top electrode. Scale bar, 10 μm. (**b**) *I-V* curves of the JJwG at various temperatures and the schematic of JJwG. (**c**) *I-V* curves of the JJwoG show an insulating behaviour similar to those of a superconductor–insulator–superconductor junction with the Josephson current completely suppressed. Schematic structure of JJwoG is shown.

**Figure 7:**
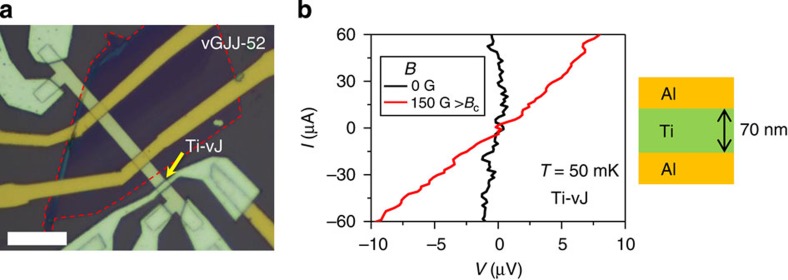
Titanium-based vertical junction. (**a**) Optical image of Ti-vJ device is denoted by an arrow. Red dotted line outlines the region of few-layer graphene. Scale bar, 10 μm. (**b**) Current–voltage (*I*–*V*) curves of the Ti-vJ measured in zero magnetic field, *B* (black curve), and *B*=150 G, which is larger than the critical magnetic field of aluminium, *B*_c_ (red curve). Schematic structure of Ti-vJ is shown on the right panel.

We clarify the role of graphene in vGJJ by comparing two different types of vertical JJs with (JJwG in Fig. 6a) and without (JJwoG in Fig. 6a) graphene insertion on the same substrate. Here, the graphene was a bilayer. The JJwG shows typical *I*–*V* curves of JJ with finite critical current and normal resistance of *R*_N_~30 Ω as shown in Fig. 6b. However, the JJwoG shows an insulating behaviour with much larger *R*_N_ ~3 kΩ (Fig. 6c). During the device fabrication processes of JJwoG, the part of Ti layer that has not been covered with graphene and exposed to the ambient environment was oxidized as shown in the schematic plot of Fig. 6c and gave degraded interfacial characteristics^32^,^33^. This implies that even the holes and defects that may have been accidently present in the graphene could not short the junction. Next, we removed the oxidized titanium layer by *in situ* Ar ion beam etching and evaporated 70-nm-thick Ti layer and 300-nm-thick Al layer in sequence, fabricating oxide-layer-free Ti-based vertical junction (Ti-vJ), shown in Fig. 7a. At the base temperature, Ti-vJ showed much smaller *R*_N_ (~0.1 Ω) and much larger *I*_c_ (>60 μA) compared with typical values of vGJJs (Fig. 7b), which indicates that the normal-metallic thin Ti layer showed a resistance too low to produce a ‘weak’-link JJ. In other words, the Ti-vJ as a whole constituted a single Al superconductor rather than a weak link JJ. In contrast, a graphene layer, although atomically thin, gave an effective weak link for the short ballistic proximity JJ. This emphasizes the uniqueness of graphene as an insertion material between superconductors for realizing the short ballistic proximity Josephson coupling.

**Figure 8:**
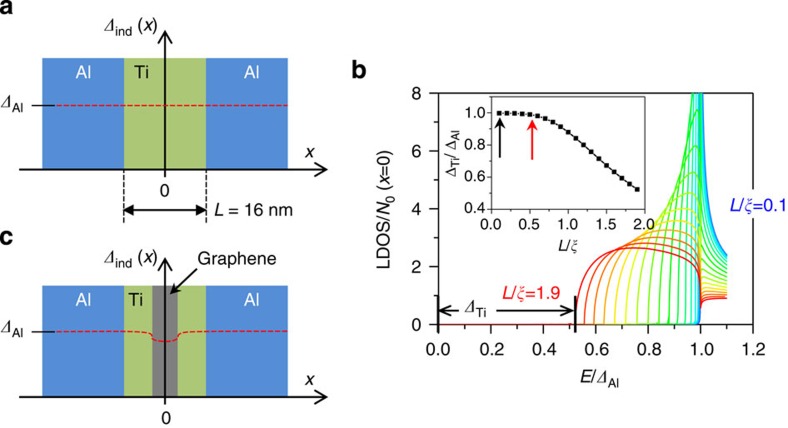
Proximity-induced superconducting gap. (**a**) Proximity-induced superconducting gap, *Δ*_ind_, is denoted by the red dashed line for aluminium (Al)/titanium (Ti)/Al JJ. *L* is the junction length between the two Al layers. (**b**) Normalized LDOS at the centre of Ti layer calculated for the structure of **a**. Inset, *L* dependence of *Δ*_Ti_=*Δ*_ind_ (0). Black and red arrows indicate the case of *L*=16 and 70 nm, respectively. (**c**) Schematic configuration of a vGJJ and *Δ*_ind_ (red dashed line).

We examine whether the Ti adhesion layer in a vGJJ should be considered as a normal metal or a superconductor by numerically calculating the proximity-induced superconducting gap Ti (*Δ*_Ti_) using quasi-classical Green’s function method. Usadel^34^ applied Eilenberger’s quasi-classical equilibrium theory^35^ to dirty superconductors adopting impurity-averaged Green’s functions. For a more convenient interpretation introduced by Nazarov^36^, these Green’s functions are parameterized by two complex angles, the polar angle 
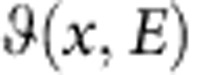
 and the azimuthal angle *ϕ*(*x*, *E*). Here *x* and *E* represent the position and energy, respectively. 
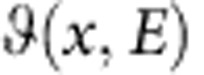
 determines the pair correlation strength and *ϕ*(*x*, *E*) represents the superconducting phase as a function of *x* and *E*. Local superconducting properties can be quantified from the solution of the Usadel’s equation:


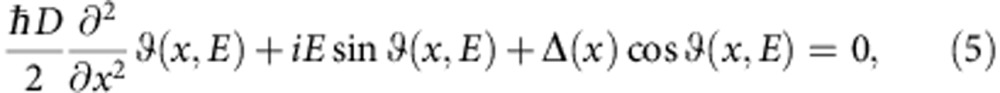


with proper boundary conditions specified at a given structure of superconducting and normal materials. Here, *D*=*v*_F_*l*/3~33 cm^2^ s^−1^ is the diffusion constant of three-dimensional normal metal with the Fermi velocity of *v*_F_~10^6^ m s^−1^ and the mean free path of *l*~10 nm for thermally evaporated Ti. *Δ*(*x*) represents the position-dependent superconducting gap of the materials themselves. For the S–N–S structure (Fig. 8a) consisting of Al and Ti without graphene layer, *Δ*(*x*) vanishes in the Ti layer but remains finite and uniform for the Al layers, that is, *Δ*(−*L*/2<*x*<*L*/2)=0, *Δ*(*x*>*L*/2 or *x*<-*L*/2)=*Δ*_Al_. *L* is the thickness of the Ti layer. By changing the variable 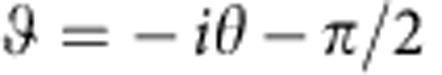, equation (5) transforms into a real second-order differential equation:


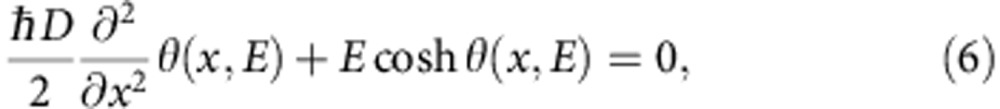


for the region of Ti layer (−*L*/2<*x*<*L*/2) with boundary conditions at the interfaces as:


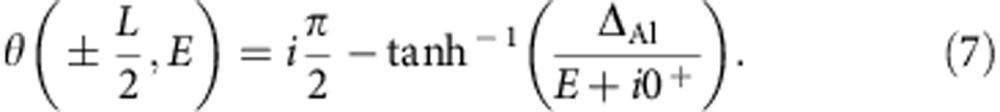


The local density of states (LDOS) at position *x* and energy *E* is given by


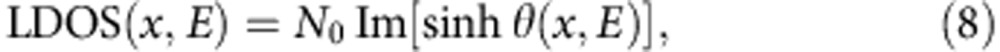


where *N*_0_ is LDOS of the normal Ti layer. LDOS at the centre of Ti layer (*x*=0) is numerically solved with varying *L* as shown in Fig. 8b. Additional scattering at the Ti/Al interfaces is neglected because of the absence of potential barrier as discussed in Fig. 5a. Superconducting coherence length, 
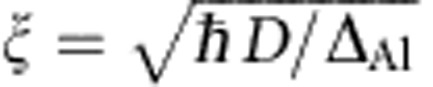
, represents the characteristic decay length of pair correlation in the normal metal. *Δ*_Ti_ is defined as a proximity-induced gap in the LDOS at the centre of the Ti layer [*Δ*_ind_ (*x*=0)] and is plotted as a function of *L* normalized by *ξ* in the inset of Fig. 8b. For the parameters of the vGJJ discussed in the main text, *L*=16 nm and *ξ*~140 nm for *Δ*_Al_=110 μeV. *L* is shorter than *ξ* by an order of magnitude so that *Δ*_Ti_ is almost identical to *Δ*_Al_. This is the case even for the Ti-vJ in Fig. 7 (*L*=70 nm) as indicated by the red arrow in the inset of Fig. 8b. Schematics of *Δ*_ind_ (*x*) are denoted by the red dashed lines in Fig. 8a,c. Ti layer has *Δ*_ind_ comparable to *Δ*_Al_, and it would result in an ill-defined ‘weak’-link JJ. This can be interpreted as a Ti layer effectively shorting the two Al superconductors. According to the analysis in Fig. 5, on the other hand, the graphene would act as a major barrier for a vGJJ.

This paper reports the first observation of Josephson coupling in a short and ballistic regime, which is expected to enable studies on exotic, but highly elusive to date, phenomena arising from strong Josephson coupling reaching the theoretical limit. The vGJJ in this study may be suited to scalable quantum devices using ABS, but further work is required to devise a way of incorporating thousands of Andreev channels present. It would also be stressed that the proposed scheme of the vertical structure is not limited to superconducting electrodes and graphene, but is readily applicable to a variety of electrodes (for example, ferromagnets) and cleavable materials with exotic properties, such as a three-dimensional topological insulating phase, layered high-*T*_c_ superconductivity of cuprates and iron-pnictides or various collective electronic states in transition-metal dichalcogenides. This will open the pathway to a wide range of research opportunities for the fundamental physics manifested at the atomic-scale interfaces of different materials, as well as the applications for highly coherent and scalable superconducting hybrid quantum devices.

## Methods

### Device fabrication

VGJJs were fabricated by the ‘flip-transfer’ scheme^16^ developed in the group, which is an extension of the ordinary graphene-transfer technique^37^. Monolayer graphene was mechanically exfoliated on a sacrificial substrate covered with a water-soluble poly (4-styrenesulfonic acid) (PSS) layer and an LOR (MicroChem) resist layer. Using standard electron (e)-beam lithography with 950 K poly (methyl methacrylate), the bottom electrode was patterned and developed. A stack of Ti/Al/Au (8/50/5 nm) was directly deposited onto graphene by e-beam evaporation. During the metal lift-off process in an 80 °C hot xylene bath, the PSS and LOR layers remained stable. After dissolving the PSS layer on the water surface to detach the sacrificial substrate, the entire structure of graphene in contact with the bottom electrode was flipped over and transferred to a new substrate. Then, the LOR layer was removed in Remover PG solution, and the top electrode (8/200/5 nm of Ti/Al/Au) was e-beam deposited onto the opposite surface of the graphene.

### Measurements

Devices were thermally anchored to the mixing chamber of a ^3^He/^4^He dilution refrigerator (Oxford Kelvinox AST) and cooled to a base temperature of *T*=50 mK. To measure the *I*–*V* characteristics, we used a four-probe configuration, shown in Fig. 1c. Current was injected between *I*+ and *I*−; the voltage difference across the junction varied from *V*+ to *V*−. All measurement lines were electrically filtered by two-stage low-pass RC filters at the mixing chamber, with a cutoff frequency of ~10 kHz, in combination with another set of RC filters and *π*-type low-pass LC filters, with a cutoff frequency of 10 MHz at room temperature. For the *T*-dependence measurements of Fig. 3b and the CPR measurements of Fig. 4c, critical switching currents were determined by averaging the critical junction currents measured repeatedly over 10^4^ times using an 18-bit data acquisition board (NI-DAQ 6281) with a linearly increasing bias current.

### Determination of the superconducting gap

**Figure 9:**
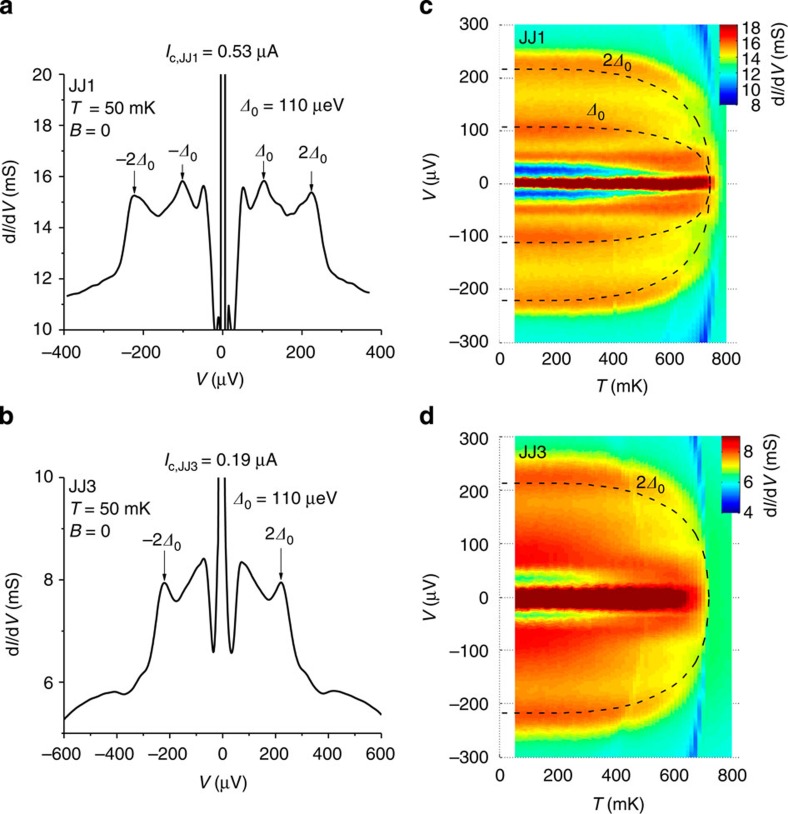
Multiple Andreev reflection. Differential conductance (d*I*/d*V*) measured at the base temperature, for JJ1 (**a**) and for JJ3 (**b**). MAR peaks are denoted by arrows. The Josephson critical current of each junction is *I*_c,JJ1_=0.53 μA and *I*_c,JJ3_=0.19 μA. The differential conductance map corresponding to the MAR as a function of temperature and the bias voltage, for JJ1 (**c**) and for JJ3 (**d**) along with the BCS-type temperature dependence of the gap voltages denoted by dotted lines.

Superconducting gap for the electrodes in our devices can be determined accurately by analysing the MAR signal. However, *I*_c_ of JJ2 (=13.3 μA) is so high that the voltage directly jumps to~200 μV after Josephson current switching (Fig. 2a and Supplementary Fig. 7), and it is not possible to observe the MAR, which appears at the subgap regime (*V*<2*Δ*_0_/*e*). However, the junctions JJ1 and JJ3 fabricated together with JJ2 using identically prepared top and bottom Al electrodes have *I*_c_’s sufficiently low as to show the MAR. There appear clear differential conductance (d*I*/d*V*) peaks at *V*=2*Δ*_0_/*e* and *Δ*_0_/*e* for JJ1 and at *V*=2*Δ*_0_/*e* for JJ3 (Fig. 9a,b). Temperature dependence of *Δ*_0_ is well described by BCS theory as shown in Fig. 9c,d. Both junctions show the identical *Δ*_0_, which ensures the junction-by-junction uniformity of superconducting electrodes and *Δ*_0_=110 μeV for JJ2.

### Correction of the screening effect in the CPR measurements

With the finite self-inductance *L*_ind_ of the SQUID loop, the phase relation is corrected by *δ*=*γ*+2*πΦ*_t_/*Φ*_0_, where *Φ*_t_=*Φ*_a_+*Φ*_s_ is the total magnetic flux, the sum of the applied magnetic flux threading the SQUID loop *Φ*_a_ and the screening magnetic flux, *Φ*_s_=*L*_ind_*I*_cir_, induced by the circulating screening current *I*_cir_=(*I*_c,tJJ_sin*γ*−*I*_J,vGJJ_(*δ*))/2 around the loop with self-inductance *L*_ind_. Calculation of *I*_c,SQ_ for the data fitting is done by maximizing *I*_SQ_=*I*_c,tJJ_sin*γ* + *I*_J,vGJJ_(*δ*) under the constraint of the above phase relation. With a small value of the screening parameter *β*_m_≡2*L*_ind_*I*_cir,max_/*Φ*_0_~0.19<1, the screening effect manifests itself as only a constant phase shift in the CPR measurements and does not show hysteresis between the curves obtained for opposite directional magnetic field sweeps. The experimental data show no hysteresis, either, in the magnetic field sweep direction. Here, *L*_ind_~7.9 pH is calculated using the commercial package COMSOL Multiphysics; *I*_cir,max_=(*I*_c,tJJ_+*I*_c,vGJJ_)/2 is the maximum circulating screening current.

## Author contributions

G.-H.L. and H.-J.L. conceived the idea and designed the project. G.-H.L. fabricated the devices and carried out the experiments. G.-H.L. and H.-J.L. analysed the data. S.K. and S.-H.J. carried out theoretical analysis for characterizing the G/Ti and Ti/Al interfaces. All authors contributed to the discussion and writing the manuscript.

## Additional information

**How to cite this article:** Lee, G.-H. *et al.* Ultimately short ballistic vertical graphene Josephson junctions. *Nat. Commun.* 6:6181 doi: 10.1038/ncomms7181 (2015).

## Supplementary Material

Supplementary Information Supplementary Figures 1-7, Supplementary Notes 1-7 and Supplementary References.

## References

[b1] Likharev K. K. Superconducting weak links. Rev. Mod. Phys. 51, 101–159 (1979).

[b2] Tinkham M. Introduction to Superconductivity Dover (2004).

[b3] Doh Y.-J. *et al.* Tunable supercurrent through semiconductor nanowires. Science 309, 272–275 (2005). 16002611 10.1126/science.1113523

[b4] Jarillo-Herrero P., van Dam J. A. & Kouwenhoven L. P. Quantum supercurrent transistors in carbon nanotubes. Nature 439, 953–956 (2006). 16495994 10.1038/nature04550

[b5] Heersche H. B., Jarillo-Herrero P., Oostinga J. B., Vandersypen L. M. K. & Morpurgo A. F. Bipolar supercurrent in graphene. Nature 446, 56–59 (2007). 17330038 10.1038/nature05555

[b6] Giazotto F. *et al.* A Josephson quantum electron pump. Nat. Phys. 7, 857–861 (2011).

[b7] Lee G.-H., Jeong D., Choi J.-H., Doh Y.-J. & Lee H.-J. Electrically tunable macroscopic quantum tunneling in a graphene-based Josephson junction. Phys. Rev. Lett. 107, 146605 (2011). 22107225 10.1103/PhysRevLett.107.146605

[b8] Rokhinson L. P., Liu X. & Furdyna J. K. The fractional a.c. Josephson effect in a semiconductor-superconductor nanowire as a signature of Majorana particles. Nat. Phys. 8, 795–799 (2012).

[b9] Veldhorst M. *et al.* Josephson supercurrent through a topological insulator surface state. Nat. Mater. 11, 417–421 (2012). 22344327 10.1038/nmat3255

[b10] Magnée P. H. C. *et al.* Influence of low energy Ar-sputtering on the electronic properties of InAs-based quantum well structures. Appl. Phys. Lett. 67, 3569–3571 (1995).

[b11] Heida J. P., van Wees B. J., Klapwijk T. M. & Borghs G. Critical currents in ballistic two-dimensional InAs-based superconducting weak links. Phys. Rev. B 60, 13135–13138 (1999).

[b12] Mizuno N., Nielsen B. & Du X. Ballistic-like supercurrent in suspended graphene Josephson weak links. Nat. Commun. 4, 2716 (2013). 24225412 10.1038/ncomms3716

[b13] Abay S. *et al.* High critical-current superconductor-InAs nanowire-superconductor junctions. Nano Lett. 12, 5622–5625 (2012). 23030250 10.1021/nl302740f

[b14] Goffman M. F. *et al.* Supercurrent in atomic point contacts and Andreev states. Phys. Rev. Lett. 85, 170–173 (2000). 10991186 10.1103/PhysRevLett.85.170

[b15] Della Rocca M. L. *et al.* Measurement of the current-phase relation of superconducting atomic contacts. Phys. Rev. Lett. 99, 127005 (2007). 17930546 10.1103/PhysRevLett.99.127005

[b16] Lee G.-H. & Lee H.-J. Josephson coupling realized in graphite-based vertical junction. Appl. Phys. Express 6, 025102 (2013).

[b17] Böttcher K. & Kopp T. Multichannel dc Josephson effect in ballistic point contacts. Phys. Rev. B 55, 11670–11687 (1997).

[b18] Kulik I. O. & Omel'yanchuk A. N. Contribution to the microscopic theory of the Josephson effect in superconducting bridges. JETP Lett. 21, 96 (1975).

[b19] Chrestin A. & Merkt U. High characteristic voltages in Nb/p-type InAs/Nb Josephson junctions. Appl. Phys. Lett. 70, 3149–3151 (1997).

[b20] Jung M. *et al.* Superconducting junction of a single-crystalline Au nanowire for an ideal Josephson device. ACS Nano 5, 2271–2276 (2011). 21355535 10.1021/nn1035679

[b21] Girit C. *et al.* Tunable graphene dc superconducting quantum interference device. Nano Lett. 9, 198–199 (2008). 19090696 10.1021/nl802765x

[b22] Kulik I. O. & Omel’yanchuk A. N. Properties of superconducting microbridges in the pure limit. Sov. J. Low Temp. Phys. 3, 459–462 (1977).

[b23] Jeong D. *et al.* Observation of supercurrent in PbIn-graphene-PbIn Josephson junction. Phys. Rev. B 83, 094503 (2011).

[b24] Beenakker C. W. J. & van Houten H. Josephson current through a superconducting quantum point contact shorter than the coherence length. Phys. Rev. Lett. 66, 3056–3059 (1991). 10043687 10.1103/PhysRevLett.66.3056

[b25] Bretheau L., Girit Ç. Ö., Pothier H., Esteve D. & Urbina C. Exciting Andreev pairs in a superconducting atomic contact. Nature 499, 312–315 (2013). 23868261 10.1038/nature12315

[b26] Nikolić B. K., Freericks J. K. & Miller P. Intrinsic reduction of Josephson critical current in short ballistic SNS weak links. Phys. Rev. B 64, 212507 (2001).

[b27] Kresse G. & Furthmüller J. Efficient iterative schemes for *ab initio* total-energy calculations using a plane-wave basis set. Phys. Rev. B 54, 11169–11186 (1996). 10.1103/physrevb.54.11169 9984901

[b28] Perdew J. P., Burke K. & Ernzerhof M. Generalized gradient approximation made simple. Phys. Rev. Lett. 77, 3865–3868 (1996). 10062328 10.1103/PhysRevLett.77.3865

[b29] Tkatchenko A. & Scheffler M. Accurate molecular Van der Waals interactions from ground-state electron density and free-atom reference data. Phys. Rev. Lett. 102, 073005 (2009). 19257665 10.1103/PhysRevLett.102.073005

[b30] Car R. & Parrinello M. Unified approach for molecular dynamics and density-functional theory. Phys. Rev. Lett. 55, 2471–2474 (1985). 10032153 10.1103/PhysRevLett.55.2471

[b31] Simmons J. G. Generalized formula for the electric tunnel effect between similar electrodes separated by a thin insulating film. J. Appl. Phys. 34, 1793–1803 (1963).

[b32] Chen S. *et al.* Oxidation resistance of graphene-coated Cu and Cu/Ni alloy. ACS Nano 5, 1321–1327 (2011). 21275384 10.1021/nn103028d

[b33] Dlubak B. *et al.* Graphene-passivated nickel as an oxidation-resistant electrode for spintronics. ACS Nano 6, 10930–10934 (2012). 23145543 10.1021/nn304424x

[b34] Usadel K. D. Generalized diffusion equation for superconducting alloys. Phys. Rev. Lett. 25, 507–509 (1970).

[b35] Eilenberger G. Transformation of Gorkov’s equation for type II superconductors into transport-like equations. Z. Phys. 214, 195–213 (1968).

[b36] Nazarov Y. V. Circuit theory of Andreev conductance. Phys. Rev. Lett. 73, 1420–1423 (1994). 10056788 10.1103/PhysRevLett.73.1420

[b37] Dean C. R. *et al.* Boron nitride substrates for high-quality graphene electronics. Nat. Nanotechnol. 5, 722–726 (2010). 20729834 10.1038/nnano.2010.172

[b38] Courtois H., Meschke M., Peltonen J. T. & Pekola J. P. Origin of hysteresis in a proximity Josephson junction. Phys. Rev. Lett. 101, 067002 (2008). 18764493 10.1103/PhysRevLett.101.067002

