# Supplementary material for: Ultimately short ballistic vertical graphene Josephson junctions
Source: Nat Commun. 2015 Jan 30;6:6181. doi: 10.1038/ncomms7181 (PMC4317505; doi:10.1038/ncomms7181)
Supplement: Supplementary Information — Supplementary Figures 1-7, Supplementary Notes 1-7 and Supplementary References. [file ncomms7181-s1.pdf]

# Supplementary Figures

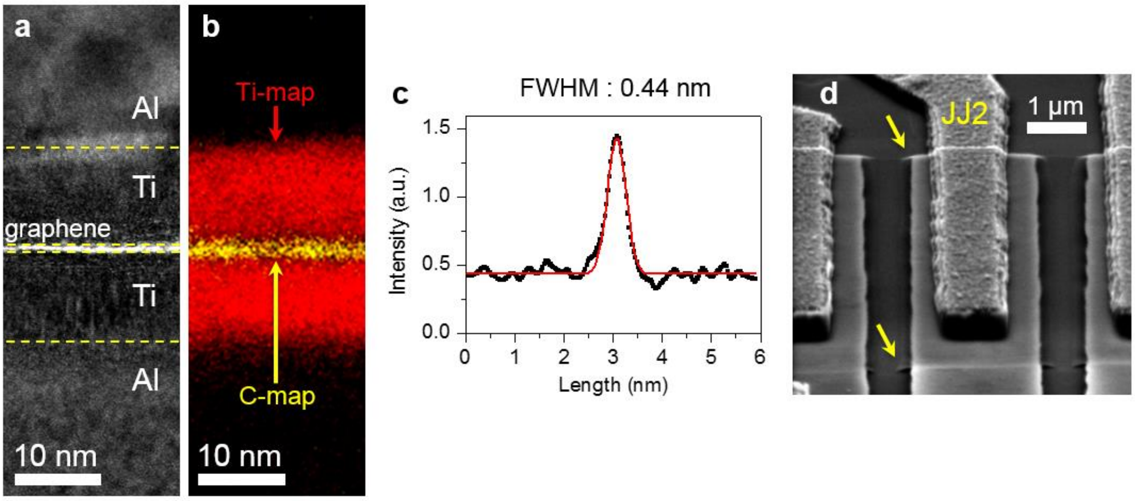


**Supplementary Figure 1 | Atomic structure of the device. a**, A HR-STEM image of cross-section of vGJJ device. **b,** An EELS image of the same area of the STEM image. Red (yellow) colour denotes the titanium (carbon) layer. The graphene (carbon) layer is sandwiched by two 8-nm-thick titanium layers. **c**, Thickness of the graphene layer is determined by a Gaussian fitting of the STEM intensity peak. **d**, An SEM image of JJ2 at a tilted angle. The monolayer graphene is not torn off and covers the bottom electrodes.

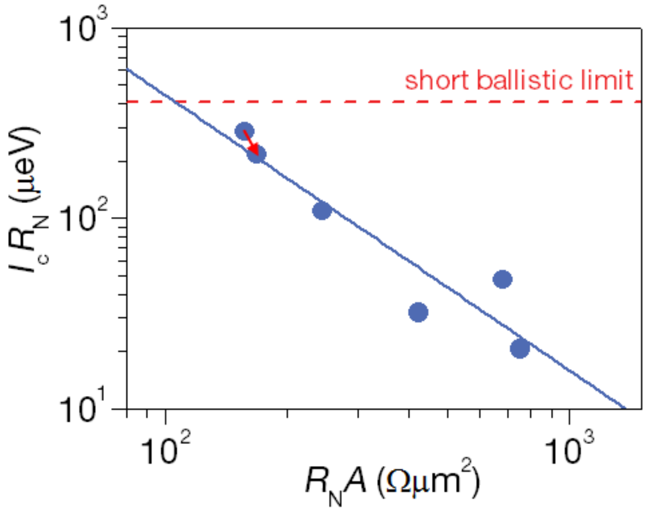


**Supplementary Figure 2 | Relation between $I_cR_N$ and $R_NA$ of monolayer graphene vGJJ.** With decreasing $R_NA$ product, $I_cR_N$ product increases approaching the theoretical limit of short ballistic regime (red dotted line). Overall trend is represented by the blue solid line. Red arrow represents the change of vGJJ (JJ2) described in the main text, after a thermal recycling.

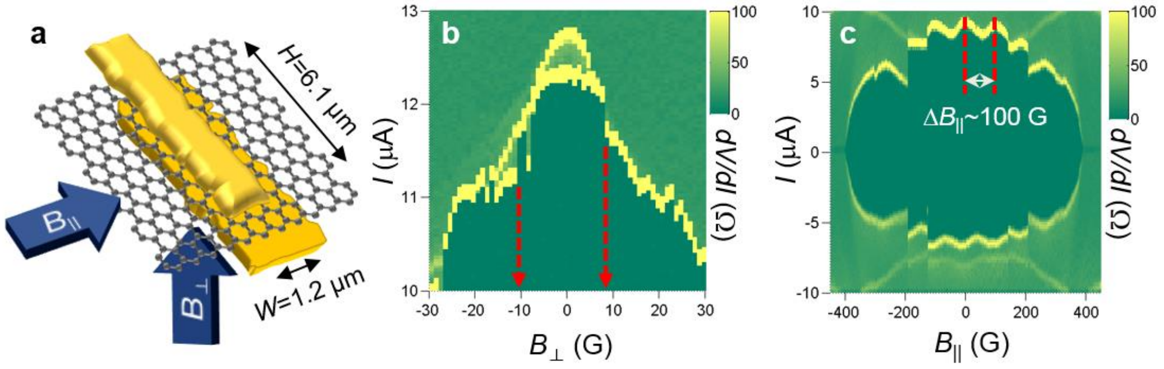


**Supplementary Figure 3 | Magnetic field dependence of $I_c$. a**, A schematic diagram showing the perpendicular ($B_\perp$) and parallel ($B_{||}$) magnetic field directions applied to the vGJJ device. **b**, Colour-coded differential resistance $dV/dI$ shows a monotonically decreasing $I_c$ with $B_\perp$. Arrows indicate sudden drops of $I_c$. **c,** The $B_{||}$ dependence of $I_c$ shows a periodic oscillation with period of $\Delta B_{||}$ ~ 100 G.

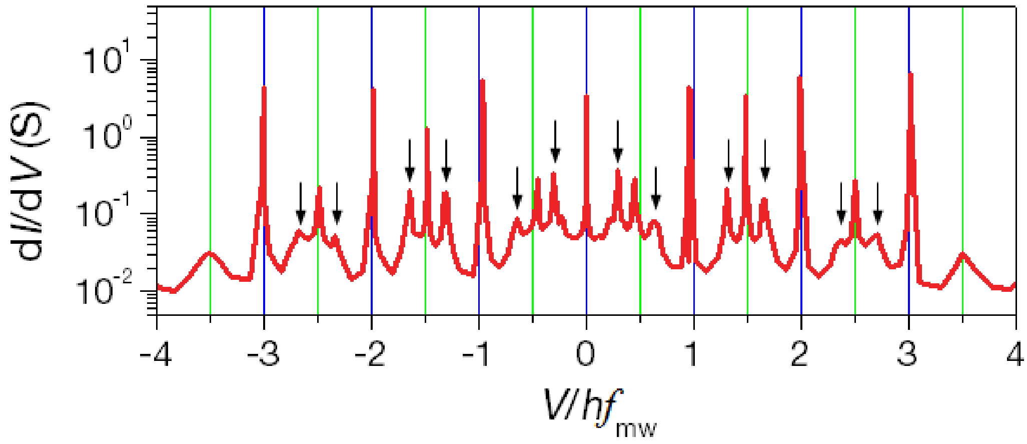


**Supplementary Figure 4 | Fractional Shapiro steps.** Differential conductance ($dI/dV$) as a function of $V/hf_{mw}$, measured at microwave frequency and microwave amplitude of $f_{mw}$ = 17 GHz and $P^{1/2}$ = 2.9 (a.u.), respectively. Peaks of $dI/dV$ represent Shapiro steps. In addition to the integer Shapiro steps (blue vertical lines), one-half (green vertical line) and one-third (black arrows) fractional Shapiro steps also appear.

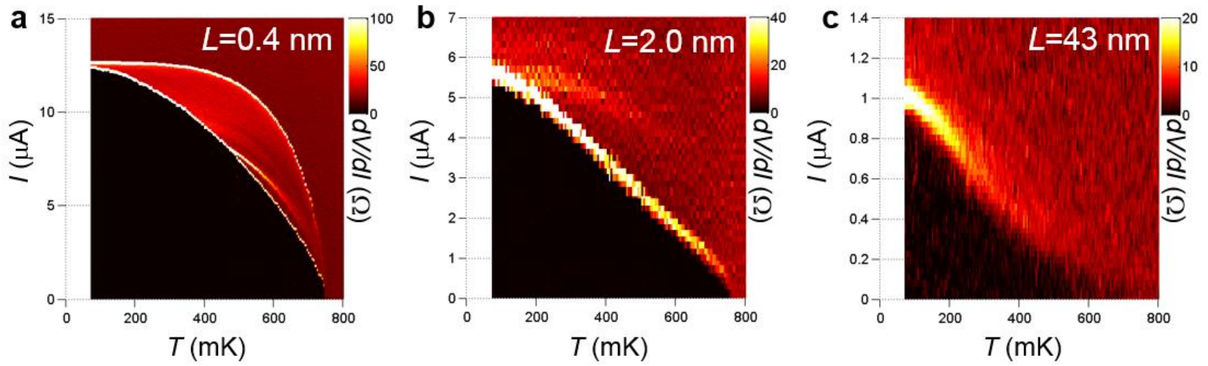


**Supplementary Figure 5 | $I_c$ vs $T$ of vertical JJs with thickness of graphite insertion. a**, A convex-shape of $I_c$ variation of a monolayer graphene vGJJ. **b**, A linear variation of $I_c$ with increasing $T$ for five-layer graphene vGJJ. **c**, A concave shape of the $I_c$ vs. $T$ curve for a 43.2-nm-thick graphite vGJJ, which represents a long diffusive junction behaviour.

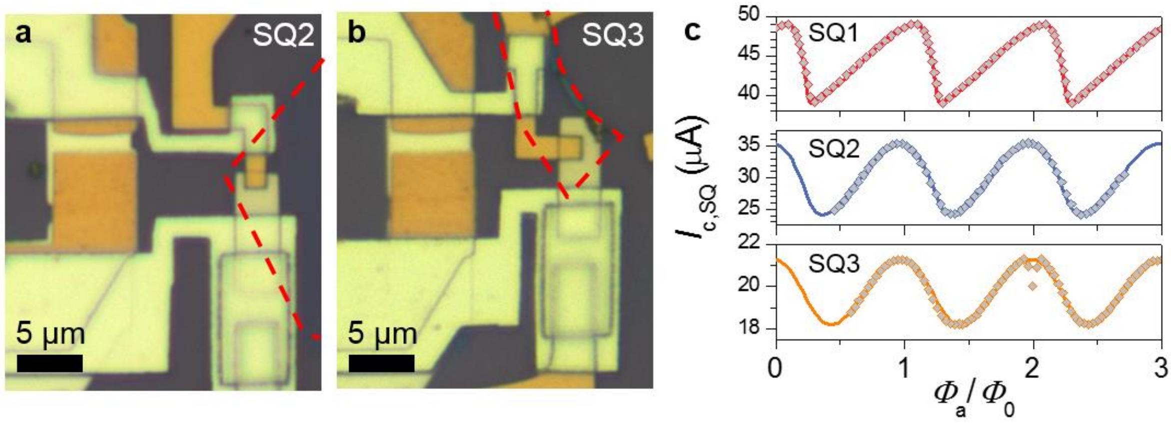


**Supplementary Figure 6 | More current-phase relation data from other vGJJs.** Optical images of dc-SQUID devices, **a**, SQ2 and **b**, SQ3 for the CPR measurements on more vGJJs. **c**, Experimentally measured $I_{c,SQ}$ (symbols) and the corresponding best-fit curves (lines). Here, SQ1 refers to the device that is discussed in the main text.

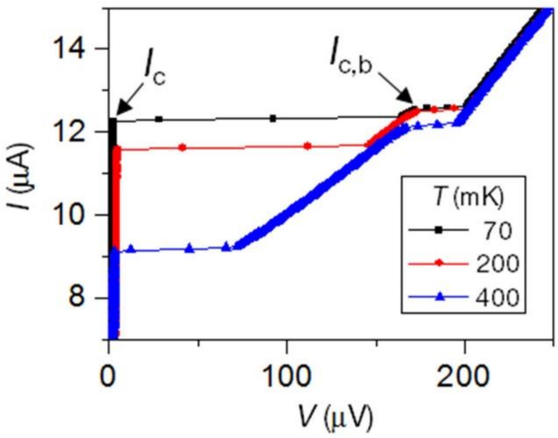


**Supplementary Figure 7 | Current-voltage characteristics of a vGJJ.** IVCs of the junction JJ2 show two different critical values, denoted as $I_c$ and $I_{c,b}$. Before the potential difference across the junction reaches the twice of the averaged gap voltage of each electrode, $2\Delta_0/e$ ~ 220 μV, the bottom electrode becomes normal, giving $I_{c,b}$.

# Supplementary Notes

## Supplementary Note 1

## Atomic structure of the device

We analysed the atomic structure of vertical graphene Josephson junction (vGJJ) devices using high-resolution scanning transmission electron microscope (HR-STEM) with image Cs-corrector (Jeol JEM-2200FS). We also determined the constituent atomic elements using a mapping of electron energy loss spectrometry (EELS). Supplementary Figures 1a and 1b show the bright-field STEM image and EELS image of the same area. Red and yellow colours map titanium atoms and carbon atoms, respectively. As shown in Supplementary Figure 1c, thickness of the graphene was estimated by fitting the STEM intensity (black dots) across the graphene interface with a Gaussian distribution (red line), giving a full-width-half-maximum (FWHM) of 0.44$\pm$0.01 nm, in good agreement with the thickness of a monolayer grapheme sheet (0.4 nm). This indicates that the vGJJ was fabricated as designed; the monolayer graphene (carbon) is atomically sandwiched between the 8-nm thick top and bottom titanium adhesion layers followed by the aluminum layers. Supplementary Figure 1d shows scanning electron microscope (SEM) image of a vGJJ (JJ2) described in the main text at a more tilted angle to show what the graphene is like at the sharp edges of an electrode. A single-atomic layer of graphene is not torn off at the sharp edges of the electrodes as indicated by the arrows.

## Supplementary Note 2

## $R_N$ dependence of $I_cR_N$ product

We have fabricated and measured several vGJJs of monolayer graphene. The transparency and normal-state resistance of the interface between graphene and superconducting electrode materials vary from sample to sample, which may depend on various parameters; such as the vacuum level and the condition of the metallic electrode deposition chamber, deposition rate and the type of adhesion metal[1] and the superconducting material, residues of e-beam resist polymer on the surface of graphene, or possible mechanical stress imposed during the transferring processes. In our vGJJ geometry $R_N$ corresponds to the sum of the normal-state contact resistance ($R_{N,C}$) and the intrinsic vertical

resistance of monolayer graphene ($R_{N,MLG}$), which has never been studied previously. Nonetheless, the $R_{N,MLG}A$ product ($A$ is the lateral area of the junction), where sample-specific geometrical factors are eliminated, can be assumed to be constant for all vGJJs fabricated in the same fashion. Therefore, we can infer the value of $R_{N,C}$, which characterises the quality of the interface, from the $R_NA$ product subtracted by a presumably constant offset of $R_{N,MLG}A$, where $R_{N,C}A = R_NA - R_{N,MLG}A$. As shown in Supplementary Figure 2, the value of the $I_cR_N$ product, which represents the strength of the Josephson coupling, increases approaching the short ballistic limit (red dotted line) with decreasing $R_NA$ product. Here, a smaller value of $R_NA$ corresponds to a reduction of $R_{N,C}$ with more ideal contact. In our junctions, Josephson coupling is not observed even at the base temperature when the value of $R_NA$ product exceeds ~1800 $\Omega\mu m^2$.

## Supplementary Note 3

## Fraunhofer pattern

We applied magnetic field, $B$, perpendicular to or in parallel with the junction as depicted in Supplementary Figure 3a. For perpendicular magnetic fields ($B_\perp$) the critical current $I_c$ decreases monotonically as the field strength increases (see Supplementary Figure 3b). We observed a sudden drop of $I_c$ at $B_\perp$~ ±10 G, which corresponds to the threshold magnetic field $B_{th} \sim \Phi_0 / (HW) \sim 3$ G, above which Abrikosov vortices are introduced on the graphene and accordingly additional dissipation is induced. Here, $\Phi_0 = h/2e$ is magnetic flux quantum, $h$ is Plank constant, and $e$ is electron charge. $H$ and $W$ define the lateral size of the junction. Bulk aluminum shows the type-I superconductivity, whereas, an aluminum thin film with thickness (~0.2 μm) less than the bulk coherence length (~ 1 μm) can support Abrikosov vortices[2]. We changed the magnetic field direction from perpendicular to parallel by warming up and recooling down the device, while the sample holder was changed. During this thermal recycling the junction was degraded and $I_c$ decreased to ~9 μA. In contrast to the case of perpendicular magnetic fields, the parallel magnetic field ($B_{||}$) dependence of $I_c$ shows a periodic modulation with period $\Delta B_{||}$ ~ 100 G as shown in Supplementary Figure 3c, which is known as Fraunhofer pattern. Period is well described by the expected value of $\Phi_0/HL_{eff}$ ~ 100 G, where $L_{eff} = L + 2\lambda_L$ is the effective channel length with the physical channel length $L$

= 0.4 nm and the London penetration depth for aluminum[3] $\lambda_L$ ~ 16 nm. We consider Ti layers as parts of superconducting electrodes since the thickness of Ti layers is much smaller than the superconducting coherence length $\xi$ ~ 140 nm in the Ti layers. Exact shape of $I_c$ modulation with $B_{||}$ is somewhat different from the standard Fraunhofer pattern. This may be explained by the presence of corrugations[4-7] in the graphene which was caused by the roughness of the substrate (spin-coated LOR). Moreover, the roughness of evaporated poly-crystalline Ti layers in contact with graphene layer would be an order of a few angstroms. Due to randomly distributed corrugations in graphene and atomic roughness of Ti layers, magnetic field $B_{||}$ is not strictly in parallel with the junction plane[8], resulting in the $I_c$ modulation deviated from the standard Fraunhofer patterns[9]. There are also sudden drops of $I_c$ near $B_{||}$ ~ ±200 G, which can be explained by misalignment of the in-plane magnetic field $B_{||}$ with respect to the junction plane. With a misalignment of 3°, $B_{||}$ ~ ±200 G will give a perpendicular component of $B_{\perp} = B_{||}\sin(3^{o})$ ~ ±10 G, at which magnetic Abrikosov vortices start to penetrate into the junction.

## Supplementary Note 4

## Fractional Shapiro steps

The conventional integer Shapiro steps appear at the voltage $V = nhf_{mw}$ with integer $n$ as indicated in the Fig. 2d in the main text. A closer inspection of Fig. 2d also exhibits fractional Shapiro steps between the integer ones, which are more evident in the plot of differential conductance ($dI/dV$) as a function of $V$ normalised by $hf_{mw}$ as shown in Supplementary Figure 4. In addition to integer Shapiro steps (blue vertical lines), one-half and one-third fractional Shapiro steps appear as peaks of $dI/dV$ indicated by green vertical line and black arrows, respectively. The observation of fractional Shapiro steps is an indirect evidence for the skewed CPR in our vGJJ. Shapiro steps reflect the CPR of JJs as they are the consequence of beating between an external microwave field and corresponding oscillating Josephson current. For a highly transparent JJ, such as an atomic point contact JJ and our vGJJ, higher harmonics of non-sinusoidal CPR were theoretically predicted to result in fractional Shapiro steps[10,11].

## Supplementary Note 5

## Graphene thickness dependence of junction characteristics

We fabricated a vGJJ out of graphene of various thicknesses to investigate the thickness dependence of the junction characteristics. Supplementary Figures 5a, b, and c show the temperature dependences of differential resistance $dV/dI$ of vGJJs fabricated with monolayer graphene, five-layer graphene, and 43.2-nm-thick graphite, respectively. The black regions represent the regions of Josephson current characteristics, $dV/dI = 0$. As discussed in the main text, a monolayer graphene device shows a convex-shape of $I_c$ variation with increasing $T$ up to the critical temperature ($T_c$) of the Josephson junction, representing the short ballistic nature (see Supplementary Figure 5a). As the graphene flake becomes 5 times thicker, the convex shape of $I_c$ variation gets flattened so that $I_c$ decreases almost linearly with $T$ as shown in Supplementary Figure 5b. In the far thicker graphite device, $I_c$ decreases with a concave-shape tail near $T_c$ of the Josephson junction, which is typical of a long diffusive junction[12] (see Supplementary Figure 5c).

## Supplementary Note 6

## Measurements of current-phase relation

Current-phase relation (CPR) is measured in two more vGJJ devices embedded in dc-SQUID interferometers. Supplementary Figures 6a and b show optical images of SQ2 and SQ3 devices for CPR measurements. Red dotted lines represent monolayer graphene. In all SQUID devices including SQ1, which is the device discussed in the main text, aluminum-based tunnelling Josephson junctions are designed to have much larger Josephson current ($I_{c,tJJ}$) than that of vGJJs ($I_{c,vGJJ}$). Experimental data of the critical current of SQUID ($I_{c,SQ}$) modulation with magnetic fields are represented by symbols in Supplementary Figure 6c and fitted to the theoretical expectation for the junction transparency of $\tau_{SQ1} = 1.00$, $\tau_{SQ1} = 0.7$ and $\tau_{SQ3} = 0.4$ as fitting parameters. The theoretical calculation was done with Eq. (1) and the phase relation described in the main text with the self-inductance of the superconducting loop taken into account. $I_{c,SQ}$ modulation which represents the CPR of a vGJJ becomes more symmetric and sinusoidal with decreasing the transparency.

## Supplementary Note 7

## Junction transparency versus excessive current

Junction transparency $\tau$ can be estimated from the excessive current $I_{exc}$, which is an offset current value of the extrapolated line of the IVCs in a high bias voltage region[13] ($V >> 2\Delta_0 \sim 220$ μeV). However, before $V$ reaches $2\Delta_0$, the critical current of bottom electrode ($I_{c,b} \sim 13$ μA) is reached as shown in Supplementary Figure 7 so that the Josephson coupling is broken. We suspect that $I_{c,b}$ as well as the critical current of the top electrode ($I_{c,t} \sim 30$ μA) is significantly reduced by the self-heating after resistive switching of the Josephson junction. In contrast to planar junction geometry, the Joule heat generated in the vGJJ cannot be effectively relaxed into the substrate but diffuses only to the electrodes so that the self-heating effect becomes more significant. Therefore, estimation of $\tau$ out of $I_{exc}$ is not possible for our vGJJ devices.

# Supplementary References


1 Giovannetti, G. *et al.* Doping graphene with metal contacts. *Phys. Rev. Lett.* **101**, 026803 (2008).

2 Tinkham, M. Effect of fluxoid quantization on transitions of superconducting Films. *Phys. Rev.* **129**, 2413-2422 (1963).

3 Kittel, C. *Introduction to Solid State Physics*. (John Wiley & Sons, Hoboken, 2005).

4 Meyer, J. C. *et al.* The structure of suspended graphene sheets. *Nature* **446**, 60-63 (2007).

5 Ishigami, M., Chen, J. H., Cullen, W. G., Fuhrer, M. S. & Williams, E. D. Atomic structure of graphene on $SiO_2$. *Nano Lett.* **7**, 1643-1648 (2007).

6 Fasolino, A., Los, J. H. & Katsnelson, M. I. Intrinsic ripples in graphene. *Nature Mater.* **6**, 858-861 (2007).

7 Geringer, V. *et al.* Intrinsic and extrinsic corrugation of monolayer graphene deposited on $SiO_2$. *Phys. Rev. Lett.* **102**, 076102 (2009).

8 Lundeberg, M. B. & Folk, J. A. Rippled graphene in an in-plane magnetic field: effects of a random vector potential. *Phys. Rev. Lett.* **105**, 146804 (2010).

9 Alidoust, M., Sewell, G. & Linder, J. Non-Fraunhofer interference pattern in inhomogeneous ferromagnetic Josephson junctions. *Phys. Rev. Lett.* **108**, 037001 (2012).

10 Cuevas, J. C., Heurich, J., Martín-Rodero, A., Yeyati, A. L. & Schön, G. Subharmonic Shapiro steps and assisted tunneling in superconducting point contacts. *Phys. Rev. Lett.* **88**, 157001 (2002).

11 Duprat, R. & Yeyati, A. L. Phase diffusion and fractional Shapiro steps in superconducting quantum point contacts. *Phys. Rev. B* **71**, 054510 (2005).

12 Dubos, P. *et al.* Josephson critical current in a long mesoscopic S-N-S junction. *Phys. Rev. B* **63**, 064502 (2001).

13 Flensberg, K., Hansen, J. B. & Octavio, M. Subharmonic energy-gap structure in superconducting weak links. *Phys. Rev. B* **38**, 8707-8711 (1988).
